# Livedoid vasculopathy – A diagnostic and therapeutic challenge

**DOI:** 10.3389/fmed.2022.1012178

**Published:** 2022-10-03

**Authors:** Maria Rosa Burg, Carolin Mitschang, Tobias Goerge, Stefan Werner Schneider

**Affiliations:** ^1^Department of Dermatology and Venereology, University Medical Center Hamburg Eppendorf, Hamburg, Germany; ^2^Department of Dermatology, University Hospital of Muenster, Muenster, Germany

**Keywords:** livedo, *Livedo racemosa*, *Livedo reticularis*, vasculopathy, livedoid vasculitis, vasculitis, thrombosis

## Abstract

Livedoid vasculopathy is a rare, chronic-recurrent occlusive disorder in the microcirculation of dermal vessels. The clinical appearance is characterized by *Livedo racemosa*, painful ulceration, located in the distal parts of the lower extremities, followed by healing as porcelain-white, atrophic scars, the so-called *Atrophie blanche*. Different conditions that can promote a hypercoagulable state, such as inherited and acquired thrombophilias, autoimmune connective-tissue diseases and neoplasms, can be associated with livedoid vasculopathy. Therefore, livedoid vasculopathy is currently considered to be a coagulation disorder, clearly distinguished from inflammatory vasculitis. Although there are hints to hypercoaguability and secondary inflammation, pathophysiology is not completely understood. Diagnosis is made by synopsis of history, clinical and histopathological findings. Early and adequate therapy is essential to maintain life quality and avoid irreversible complications. Better understanding of molecular mechanisms is required to establish appropriate therapy regimens. This article presents the current state of knowledge about livedoid vasculopathy and proposes an algorithmic approach for diagnosis and therapy.

## Introduction

Livedoid vasculopathy (LV) is a rare, chronic-recurrent, thrombo-embolic disease with occlusions in dermal vessels, especially on the lower extremities (1).

In the literature, different names for this disease were introduced. First described as *atrophy blanche* by Milian et al. (2), Feldaker et al. used the term *Livedo reticularis with summer ulceration* 1956 (3). Milstone et al. focussed on the clinical appearance with *Painful Purpuric Ulcers With Reticular Patterning on the Lower Extremities (PURPLE)* (4). Bard and Winkelmann (5) established the term *Livedo vasculitis* and suggested that LV was a segmental hyalinizing form of vasculitis (5). Also findings in direct immunofluorescence with deposition of fibrin, immunoglobulins, and complement components localized to the hyalinized vessel walls were initially misinterpreted as a consequence of primary vasculitis (6).

However, normal serum-complement levels, a diffuse homogenous instead of granular deposition pattern, a slight perivascular infiltration of leukocytes and the absence of nuclear dust (so called leukocytoclasia) argue against an immunocomplex-mediated disease like inflammatory vasculitis (7, 8). The characteristic intraluminal thrombi as well as the response to anticoagulation therapy support the theory that thrombotic or microcirculatory mechanisms might be acting in the pathogenesis of LV, not vasculitis, so that McCalmont, Jorizzo and colleagues first proposed the term *livedoid vasculopathy* in 1992 (3, 7).

Although LV is an orphan disease, it can be very limiting for the affected patient’s quality of life (9). Occluding vasculopathy in the dermal vessels lead to ischemia and so massive pain (9, 10). Early and correct diagnosis as well as adequate therapy is important to prevent acute ischemia and so long-term consequences such as a chronic pain syndrome and dysaesthesia.

In this review, we give an overview of the current state of literature about LV, provide a diagnostical and therapeutical approach and distinguish LV from other differential diagnoses, especially inflammatory vasculitis.

## Methods

### Search strategy

This systematic review was conducted according to the Preferred Reporting Items for Systematic Reviews and Meta-Analyses (PRISMA) guideline (11).

From March of 2022 until July of 2022 a structured literature search was performed on PubMed and Cochrane. The final literature search was performed on 31 July 2022. Inclusion criteria comprise the key words “livedoid vasculopathy” AND/OR “livedoid vasculitis” English or German full text and content appropriate to the investigated topic. No other filters or tools were used. Studies without a definite diagnosis of LV were excluded. All abstracts and included full texts were reviewed by the first author.

### Study selection

**FIGURE 1:**
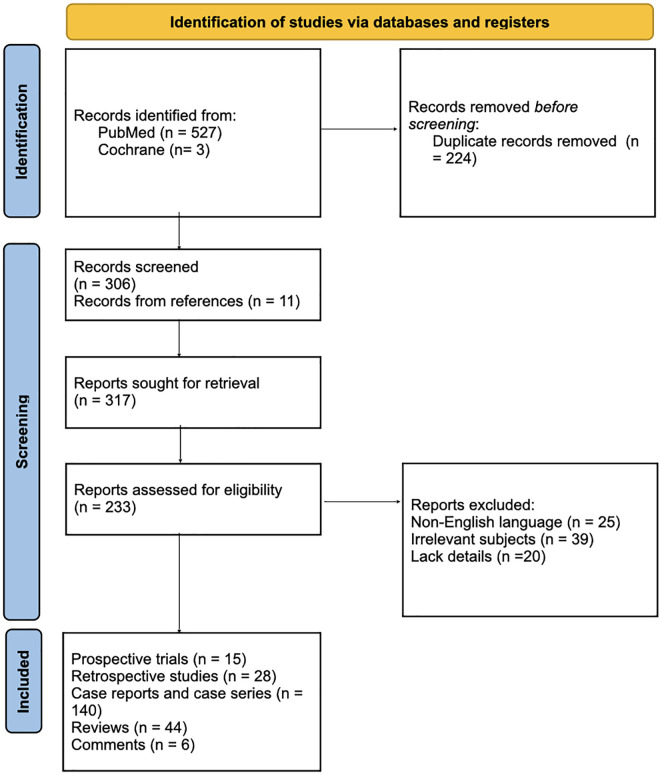
PRISMA 2020 flow diagram for new systematic reviews which included searches of databases and registers only. From Page et al. (72).

The first search process showed 527 articles from the PubMed and Cochrane online electronic databases, removing 224 duplicated records. Another 11 articles were noticed from references of the screened articles. The first author screened the abstracts of the identified articles and selected records for full-text view. The PRISMA flow diagram represents the process of literature search and study selection (Figure 1).

Inclusion criteria comprise (1) articles about LV or LV mentioned in the text; (2) article types including research articles, reviews, case series, case reports and correspondences; and (3) articles in english or german language. Exclusion criteria were as follows: (1) Articles containing information that is not matching to LV; (2) articles written in languages other than English or German. (3) No further information available. Therefore, 84 records were excluded because of the following reasons: (1) not published in english or german language (*n* = 25); irrelevant subjects (*n* = 39); and (3) lack of further details (*n* = 20).

The risk of bias is assessed to the Cochrane handbook of systematic reviews of interventions. We did not only enroll research articles, but also reviews, case series and case reports. Although this could lead to overreporting and overestimation of particular results, we did not want to renounce this information due to the rareness of the disease and the limited evidence.

## Results

### Epidemiology

LV is an orphan disease with an estimated incidence of 1:100,000, often affecting young to middle-aged women with a median age of 32 years up to 53 years in other study populations (1, 12, 13). The female-to-male ratio is described between 2.1:1 (12, 13) to 3:1 (14). In greater patient populations few pediatric patients were mentioned (15, 16).

Criado and colleagues recently reported in the context of a study on 75 patients with LV in Brasil, that the most affected age group was between 20 and 48 years with a median age of 34.7 years (17). Interestingly, 14 out of 75 patients (18.7%) were under 18 years old at the beginning of the disease (17). Similarly, Feng et al. described a very young patient population from china with 24 patients with LV with a median age of 17.0 years at beginning of the disease (18).

Although incidence is often indicated as 1:100,000 in the literature, as LV maybe unfamiliar to many physicians and in regard to our own experience, we assume a much higher incidence. Some studies report a delay from first symptoms to diagnosis of median 6.65 years, ranging from 1 to 20 years (17).

### Pathophysiology

The pathomechanism of LV is not completely understood at the current state. Initially LV was considered as vasculitis (19). Up to date LV is seen as a vascular disease with a domination of procoagulatory factors leading to a status of hypercoagulability (14). The thrombotic effect possibly results from defects in endothelial dysfunction such as impaired plasminogen activation, dysfunction of platelets or increased or restricted fibrin formation or lysis, respectively. Fibrin deposition and thrombus formation act as a diffusion barrier and lead to a decreased oxygen supply with subsequent necrosis (=skin infarction) (14, 20). Moreover, slight tissue perfusion leads to poor wound healing – a vicious circle develops (21). Hypercoagulability, stasis and endothelial damage, the so-called Virchow trias, also act as risk factors for microvascular thrombosis in LV (20, 22). Lower concentration of thrombolytic factors as well as differences in perfusion pressure and in temperature are supposed to be reasons for the manifestation of LV on the lower extremities (22, 23).

Associations to various diseases linked to hypercoagulability in patients were described in LV, including hereditary and acquired thrombophilias (e.g., Faktor V Leiden-mutation, protein C- and protein S-deficiency, antithrombin-III-deficiency, prothrombin G20210A-mutation, plasminogen-activator-inhibitor-1(PAI-1)-promoter-mutation, lipoprotein(a) (Lp(a)), methylentetrahydrofolat(MTHFR)-gene-mutation, homocysteinemia, antiphospholipid-antibodies), autoimmune diseases (e.g., systemic lupus erythematodes) and malignancies (14, 24).

Recently, a systematic review about genetic variants in LV by Gao et al. showed, that *PAI*-*1* -675 4G/5G was the most common genetic variant, accounting for 85% of 95 LV (81/95) patients analyzed. Further genetic variants comprise PAI-1 A844G (56% of 18 LV patients), MTHFR C677T and MTHFR A1298C variants (55% of 129 LV patients and 44% of 82 LV patients, respectively) (24). Less frequent findings were Factor V G1691A and Prothrombin G20210A polymorphism in 14% out of 135 and 11% out of 85 LV patients, respectively. Genetic variants differed depending on geographical and ethnical factors (24).

Among the mentioned thromophilic factors identified in LV patients, attention should be paid to Lp(a).

Lp(a) represents an independent, genetically determined and not life-style-driven risk factor for cardiovascular diseases and was frequently detected to be increased in patients with LV (25). It has a lipid core of Low Density Lipoprotein (LDL)-cholesterol bound to an apoB-100 particle, connected to the glycoprotein apolipoprotein(a) (Apo(a)) by a disulphide bridge. Apo(a) has structural similarity with plasminogen (25). It is presumed that Lp(a) has pro-thrombotic and anti-fibrinolytic properties due to (1) competition with plasminogen and consecutive impairment of plasminogen activation and/or formation of active plasmin, (2) enhancement of PAI-1 by the oxidative form of Lp(a) (ox-Lp(a)), (3) elevation of tissue factor (TF)-expression and (4) blocking of tissue factor pathway inhibitor (TFPI) (26, 27).

Criado et al. presented a greater patient cohort of 75 in Brazilian patients with LV (17). Among the 72 patients, who received a complete exam, 66% (48/72) showed associated thrombophilia factors and most of all, elevated Lp(a)-levels in 42% (30/72). The same team also reported about increased Lp(a) expression in lesional skin compared to control skin (27). Weishaupt et al. reported increased Lp(a) (42%, 5/12) and homocysteine (83%, 10/12) levels as the most frequently observed thrombophilia factors in their cohort of 25 patients (13). It must be noted that Lp(a) was only screened in a few studies.

Although a plethora of pro-thrombotic factors were identified as potential triggers of LV, most studies showed that in less than half of patients a known procoagulatory factors could be identified (Hairston et al.: 41%/12/29), Di Giacomo et al.: 52% (18/34), Weishaupt et al.: 44%/11/25), Lee et al.: 42.5% (17/40) (13, 15, 28, 29).

Only few studies showed a higher percentage of thrombophilia factors of LV patients, such as Gardette et al. with 77% (20/26) of patients with at least one positive thrombophilia factor (16) and Gao et al. with 73% (8/11) of patients (30). Gao et al. did not declare further specifications, whereas Gardette et al. again reported about hyperhomocysteinemia as most common thrombophilia factor in 50% of patients. However, prospective, well designed clinical studies involving higher patient numbers are lacking.

Because of the heterogeneity of procoagulatory factors and aetiology, LV can be divided in a primary form (idiopathic) and a secondary form on the basis of underlying other diseases (1, 31).

Besides direct participation by a hypercoagulability condition in affected vessels, there is evidence that inflammation plays a role in pathogenesis of LV. Examples are molecules like interleukins (such as interleukin-2 and soluble interleukin-2-receptor) that are released by the endothelium in the progression of the disease followed by the recruitment of leukocytes promoting inflammation (19). Even if LV is no vasculitis, the potential association to autoimmune diseases as well as the potential therapeutic respond to immunosuppressive and immunomodulatory agents, such as prednisolone, azathioprine and colchicine or intravenous immunoglobulins, suggest at least a secondary inflammatory component (14).

Furthermore, some mechanisms involved in inflammation and coagulation in patients with corona virus disease 2019 (COVID-19) show similarities with the microthrombosis detected in LV patients (32). Kyriakoudi et al. recently presented a critically ill, severe acute respiratory syndrome coronavirus type 2 (SARS-CoV-2)-positive patient with respiratory failure and livedoid skin lesions (33). Histology showed an occlusive, pauci-inflammatory vasculopathy of the cutaneous small vessels (33). Similar findings were described by Llamas-Velasco et al. (34). Perry et al. reported about a patient with secondary cryofibrinogenemia-induced LV associated with COVID-19 (35). Moreover, an exacerbation and a relapse of LV were reported after an infection with COVID-19 (36, 37).

### Histology

**FIGURE 2:**
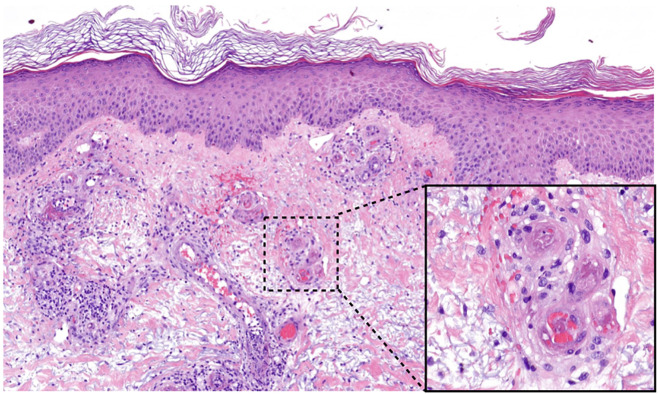
Histology of LV lesions. Fibrinoid thrombi and erythrocyte sludge in small vessels of the upper dermis with perivascular lymphocytes and extravasated erythrocytes (HE, magnification 50×, detail 400×).

Histology of skin biopsies shows fibrin deposition in the vessel walls (often difficult to recognize), endothelial proliferation and frequently intraluminal hyaline thrombi in blood vessels, especially of the upper and middle dermis, in the acute phase (Figure 2). A spare perivascular inflammatory infiltrate and leukocytoclasia maybe detected in the acute phase, but these findings are not decisive for the diagnosis (13).

If the biopsy is taken at a later stage, for example in the stage of *Atrophie blanche*, histology shows scar tissue with few vessels and an atrophic epidermis (38). In addition, a reorganization of the thrombi with subintimal proliferation and segmental hyalinization of the vessel walls and the dermis can occur (31, 39). It should be paid attention to the fact, that histological findings differ according to the stage of the disease (13).

Interestingly, in LV multiple immunoreactants, especially complement factor C3 (C3), fibrinogen and Immunoglobulin M (IgM), less Immunoglobulin A (IgA) and Immunoglobulin G (IgG), can be found in direct immunofluorescence stainings, although these findings are rated as non-specific and non-diagnostic (8, 15, 40, 41). The most reported pattern is granular deposition in the walls of blood vessels combined with or without depositions at the dermoepidermal junction (42). Positive results in the direct immunofluorescence were statistically significant more frequent in older patients and more recent lesions (<6 months) (8).

### Clinical findings

**FIGURE 3:**
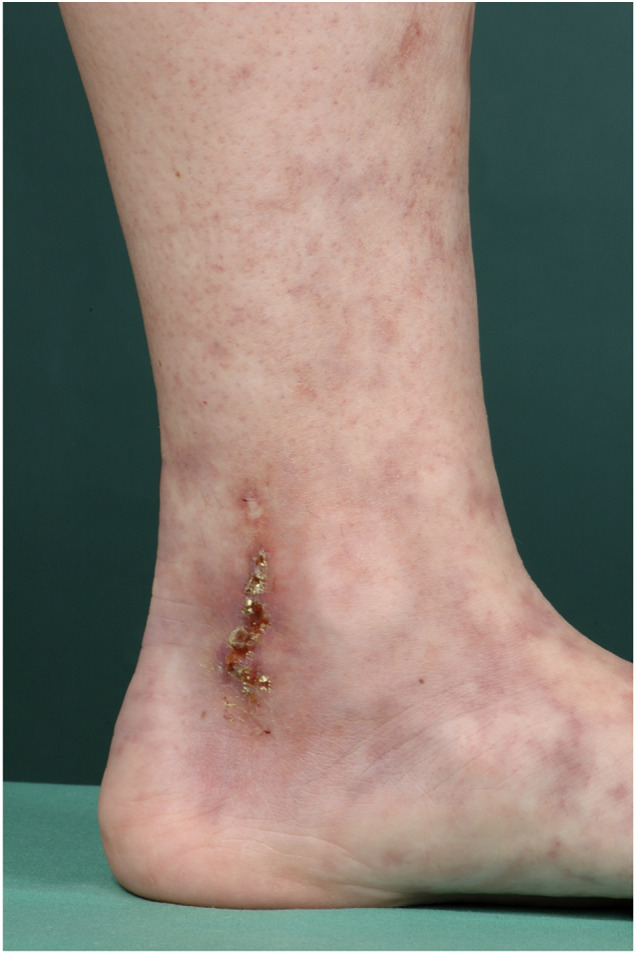
Typical presentation of LV. 33-year old female patient with typical presentation of LV: *Livedo racemosa*, ulcers and *Atrophie blanche.*

LV is characterized by the clinical trias of (i) *Livedo racemosa*, (ii) very painful ulcers followed by healing as (iii) porcelain-white scars, the so-called *Atrophie blanche* (Figure 3) (13).

*Livedo racemosa* describes a net-like red to livid coloration of the skin with discontinuous circles and reflects a pathological reduced blood flow followed by a local tissue hypoxia and ischemia. *Livedo racemosa* is not only localized on the lower extremities, but can also involve body parts above the waistline including arms, hands and back (13).

**FIGURE 4:**
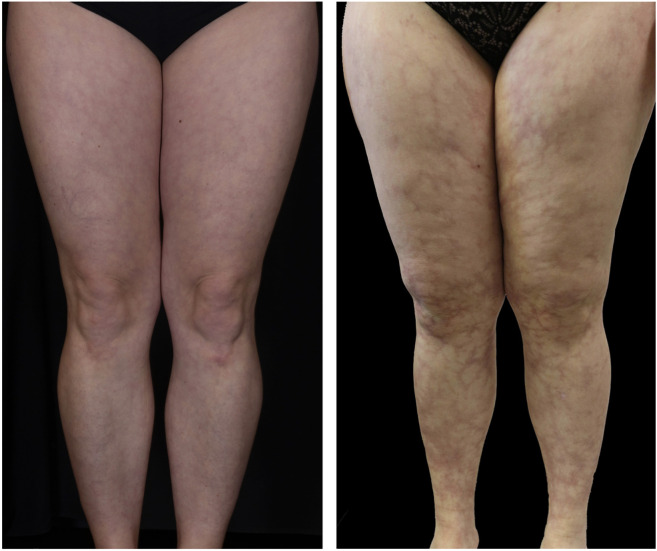
*Livedo reticularis* vs. *Livedo racemosa.*

The physiological reaction of reduced blood flow following exposure to the cold presenting as closed livid ring structures is called *Livedo reticularis* in the german-speaking community of dermatologists. Thus, *Livedo racemosa* clearly differs from *Livedo reticularis* and is characterized by irregular and open ischemic rings indicating an occlusive vascular disease such as LV (Figure 4).

Although *Livedo racemosa* can often be found in patients with LV, it is not pathognomonic and can be seen in other diseases (Table 1) (43). The in Table 1 presented disorders are mainly responsible for Livedo racemosa, but far not complete. For more details see the reviews by Georgesen et al. and Llamas-Velasco et al. (34, 44).

**TABLE 1:** Diseases associated with *Livedo reticularis* and *Livedo racemosa* (selection).

*Livedo reticularis*	*Livedo racemosa*	

Vasoconstriction	Obstacle in in-/outflow	Enhancement in viscosity
Cutis marmorata	Livedoid vasculopathy	Cryoglobulinemia
Amantadine-induced	Panarteriitis nodosa	Hematological causes (e.g., thrombocythemia)
	Antiphospholipid syndrome	Intravasal coagulation or agglutination
	Sneddon’s syndrome	
	Calciphylaxis/Martorell’s hypertensive ulcer	
	Systemic lupus erythematodes	
	Thrombangitis obliterans	
	Cholesterol embolization syndrome	
	Infectious diseases (e.g., tuberculosis, syphilis, borreliosis)	

**FIGURE 5:**
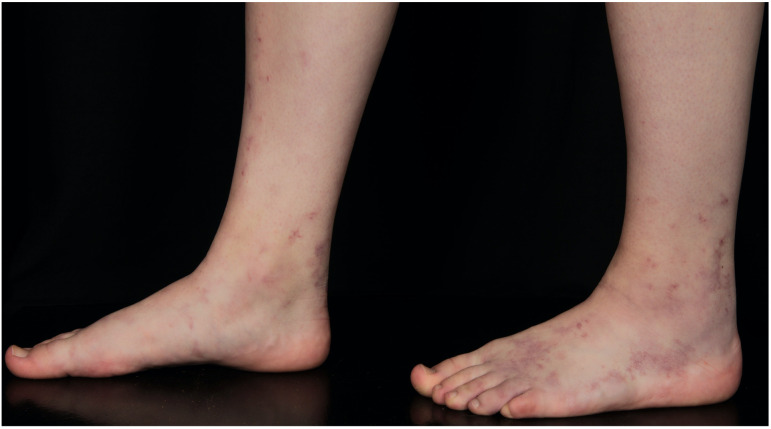
16-year old female patient with initial lesions of LV.

**FIGURE 6:**
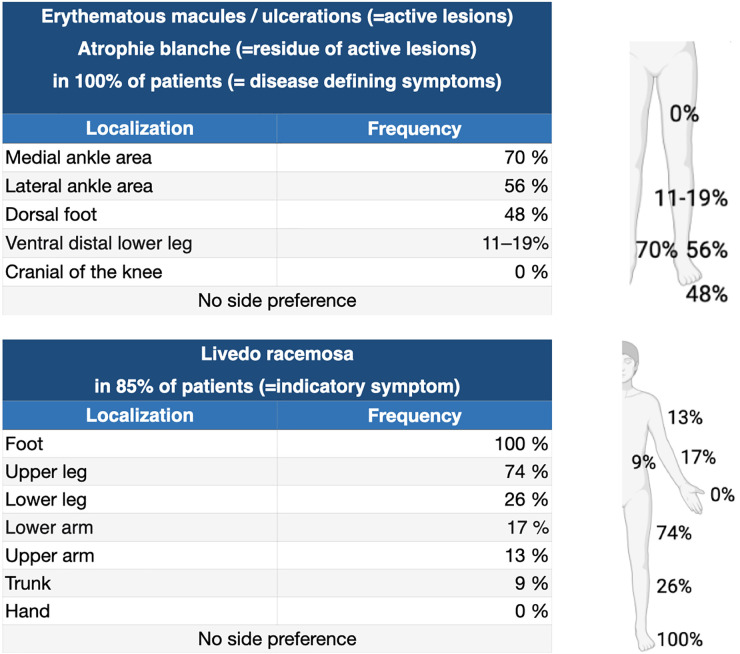
Clinical presentation of LV. Distribution of clinical symptoms of LV – adapted from Weishaupt et al. (13).

Initial lesions of LV can be flat to elevated purpuric lesions, which can ulcerate in the further course (Figure 5) (14). These ulcerations are typically sharply but bizarre defined, superficial and small (4–6 mm in diameter) and are localized on the malleolar region, dorsal feet and lower legs, but not above the knees (Figure 6; 13–15).

**FIGURE 7:**
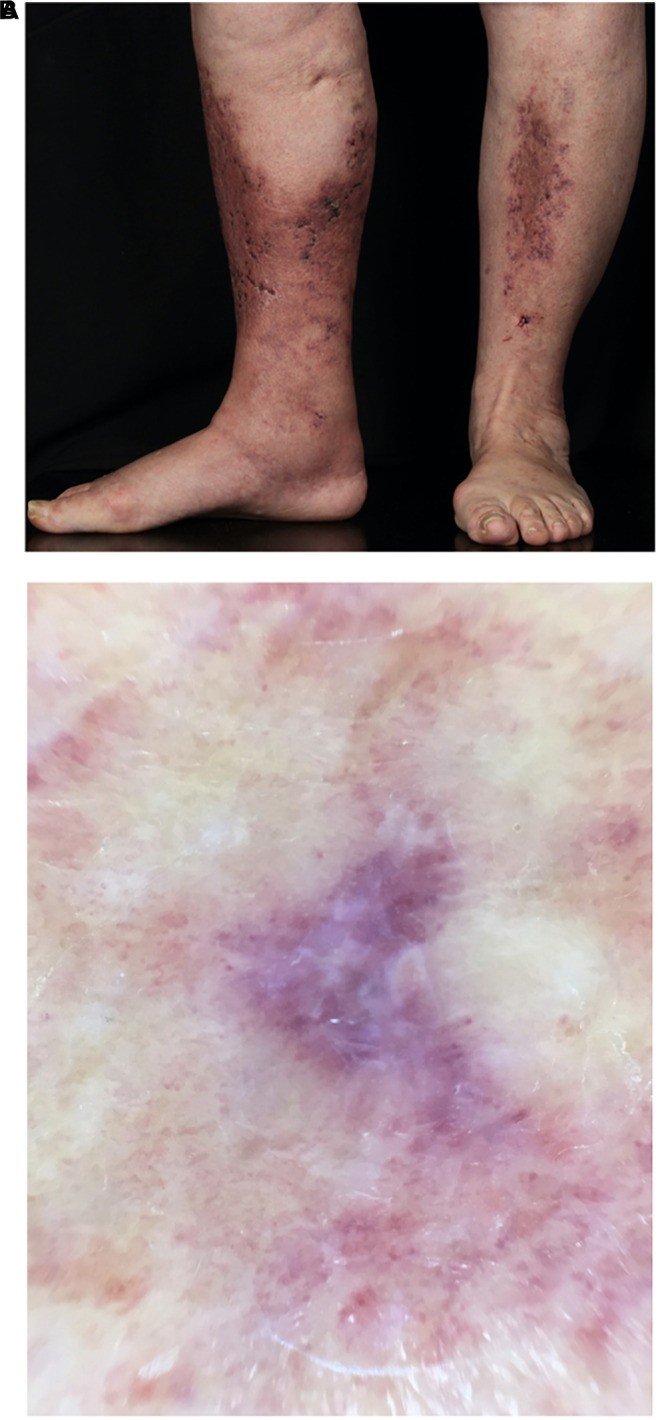
**(A)** *Atrophie blanche.* 78-year old female patient with multiple small ulcerations below the knees leaving porcellain-white, atrophic scars after healing (*Atrophie blanche*). **(B)** Dermatoscopy of LV. Ivory white scar-like areas in the center and hyperpigmentation in the periphery of the lesions.

After healing the skin remains atrophic, stellate, scar-like, porcellain-white plaques with teleangiectasia and peripheral hyperpigmentation – the so-called *Atrophie blanche* (Figure 7A; 15). Dermatoscopy shows shallow crusted ulcers and ivory white scar-like areas in the center of the lesions and hyperpigmentation in form of reticular pigmentation and increased vascular structures in the periphery of the lesions (Figure 7B; 45, 46). Histopathological correlation reveals dermal fibrosis at the center of lesions with ivory white areas in the dermatoscopic picture following the healing of the ulcers. The reticular pattern at the periphery is related to hyperpigmentation of the basal layer of the epidermis or melanin within melanophages in the dermal papillae. The vascular structures is correlated with dilatation and proliferation of capillaries in the upper dermis (45). Lesions of LV are mainly located at the malleolar region or dorsal feet with a bilateral appearance (Figure 5; 13). Although LV was first described as *Livedo reticularis with summer ulcerations* by Feldaker et al. (47), LV occurs perennial, not seasonal, even if the course of disease is often recurrent and some authors describe exacerbation during warm weather seasons (17, 38).

Some authors reported a mentionable delay from first symptoms to diagnosis from 1 to 20 years (median 6.65 years), possibly due to lack of knowledge about LV, confusion with other diseases or delayed consultation of medical centers familiar with this disease (17). Other authors described a period of 10 months from first symptoms to diagnosis and 22.5 months to beginning of treatment (13, 48). LV is characterized by intensive pain (median pain on the visual analogue scale 65⋅0) triggerd by skin ischemia due to the occlusion of dermal vessels (10). Studies show that the intermittent tormenting pain leads to a dramatical impairment of life quality (9, 10).

In a very severe form of LV patients also describe symptoms of a polyneuropathy, like dys- and hypoesthesia. Patients report of abnormal skin sensations sometimes associated with pain or hypoesthesia mainly located at the outside part of lower legs and back of the foot. These symptoms can often be associated to Mononeuritis multiplex, a peripheral neuropathy with possible associations to diabetes, neoplasms and infections. In contrast to other diseases associated with mononeuritis multiplex, where vasculitis can be seen in the vasa nervorum, in LV thrombus formation can be seen in the vasa nervorum comparable to thrombus formation in the dermal vessels (49, 50).

The most reported comorbidities in LV are systemic hypertension, obesity, type II diabetes mellitus and venous insufficiency (13, 17).

### Diagnosis

**FIGURE 8:**
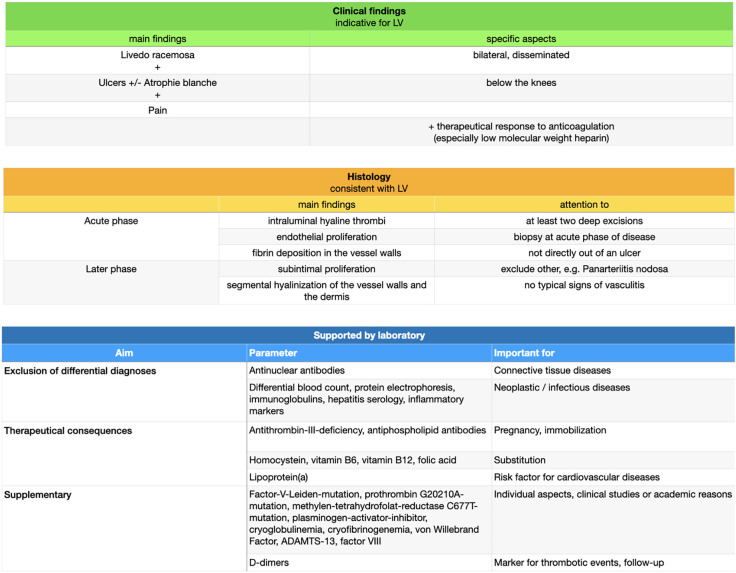
Diagnostical approach.

Even if the clinical appearance of LV is very typical (51), the diagnosis of LV should only be made in synopsis of clinical and histological findings for exclusion of differential diagnoses (Figure 8; 34, 52).

At present, there is no validated score for diagnosis of LV.

LV often affects the superficial and middle dermis, less often also the deep dermis. A deep excision in the acute stage of the disease is especially acquired to exclude other differential diagnoses affecting deeper lying areas of the skin, e.g., panarteriitis nodosa (PAN) as a vasculitis of middle-sized arteries (53). A superficial punch biopsy could probably not reach these areas and is not adequate (1, 31).

Moreover, taking tissue directly out of an ulcer should be avoided. Histology of ulcerated areas only show granulation tissue and a secondary inflammatory reaction as part of wound healing (15).

Frequently, more than one biopsy (we suggest at least two biopsies at once) is necessary to find the vascular changes typical for LV as the histopathological characteristics are segmental and not ubiquitous (15).

Detection of different laboratory parameters is especially important for distinction of differential diagnoses but has only modest therapeutical consequences for the affected patients, e.g., with regard to genetic consultation or vitamin substitution in case of hyperhomocysteinemia (31). In most cases detection of a thrombophilic factor has no therapeutical consequences and therefore a general detection of any with coagulopathies associated laboratory parameters is not recommended (54). Exceptions are antithrombin-III-deficiency and antiphospholipid antibodies (Lupus anticoagulant, Anticardiolipin antibodies IgG or IgM, Anti-beta-2-glycoprotein-I antibodies IgG or IgM) due to their therapeutical consequences during pregnancy or in case of immobilization. Moreover, we recommend analyzing homocystein, vitamins B6, B12 and folic acid as substitution can be easily performed. Finally, Lp(a) as a risk factor for cardio-vascular diseases should be measured as well as antinuclear antibodies to exclude connective tissue diseases. The german guidelines further suggest the measurements of protein C and S (38). In order to be comprehensive following thrombophilic factors or genetic analysis can be performed (i.e., due to individual aspects, clinical studies or academic reasons):

Factor-V-Leiden-mutation, prothrombin G20210A-mutation, methylen-tetrahydrofolat-reductase C677T-mutation, plasminogen-activator-inhibitor, cryoglobulinemia, cryofibrinogenemia, von Willebrand Factor, a disintegrin and metalloproteinase with a thrombospondin type 1 motif, member 13 (ADAMTS-13), factor VIII and factor IX.

To exclude a neoplastic or infectious disease a regular blood test including protein electrophoresis, immunoglobulins, hepatitis and HIV serology and inflammatory markers should be performed (31).

Please note that coagulation tests could be influenced by a thromboembolic event or under anticoagulatory therapy. Furthermore, secondary reasons for a status of hypercoagulability should be considered, like immobilization due to hospitalization, trauma or surgery, consuming diseases (e.g., malignancies), pregnancy, medicaments (e.g., anticoagulation or oral contraceptives), adipositas or advanced age (20, 54). D-dimers are indeed unspecific for a distinct disease, but represent also in LV a rapid test for thrombotic events and is increasingly seen as follow-up marker (55).

### Differential diagnoses

**FIGURE 9:**
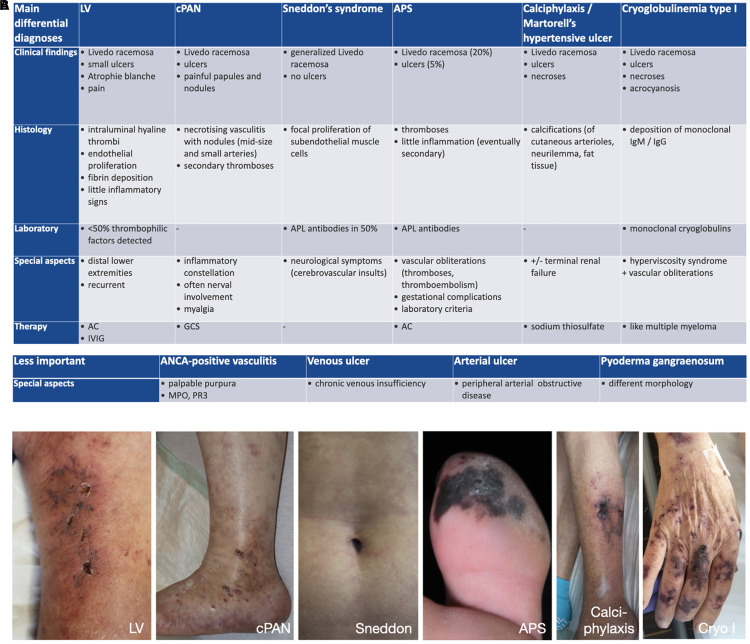
**(A)** Differential diagnoses. AC, anticoagulation; ANCA, anti-neutrophil cytoplasmatic antibodies; APL, antiphospholipid; APS, antiphospholipid syndrome; cPAN, cutaneous Panarteriitis nodosa; GCS, glucocorticosteroids; IgG, immunoglobulin G; IgM, immunoglobulin M; IVIG, intravenous immunoglobulins. **(B)** Differential diagnoses – clinical examples. LV, livedoid vasculopathy; cPAN, cutaneous Panarteriitis nodosa; APS, antiphospholipid syndrome; Cryo I, cryoglobulinemia type I.

Differential diagnoses contain cutaneous PAN, cutaneous immune-complex vasculitis, antiphospholipid-syndrome, pyoderma gangraenosum, cryoglobulinemia type I, Sneddon’s syndrome, and warfarin-induced cutaneous necrosis (Figures 9A,B; 31).

*Livedo racemosa* represents a good indication of LV. However, *Livedo racemosa* can not only be seen in LV, but in various other diseases, including PAN, Sneddon’s syndrome, antiphospholipid-syndrome, calciphylaxis, autoimmune diseases like systemic lupus erythematodes, malignancies or secondary after certain medicaments (e.g., warfarin) (43). The presence of livedo racemosa often leads to patient referrals to dermatology for exclusion of Sneddon’s syndrome. Thus far, there is no evidence that LV affects any other organ than the skin and patients with neurologic findings and LV need to be referred to neurologists for further examination.

The most important differential diagnosis is cutaneous PAN, a small- to medium necrotizing vessel vasculitis of the deep dermis and/or hypodermis. The symptoms are comparable with LV including *Livedo racemosa* and painful ulcers. However, PAN almost always shows subcutaneous nodes and signs of Mononeuritis multiplex (31). Finally, histopathological examination will distinct both entities.

Although the term *Atrophie blanche* is sometimes used as a synonym for LV, atrophie-blanche-like scars can also be seen in other diseases, e.g., chronic venous insufficiency, antiphospholipid-syndrome, cutaneous immune complex-vasculitis, systemic lupus erythematodes and sclerodermia, and is not pathognomonic for LV (1, 52, 56).

### Therapy

**FIGURE 10:**
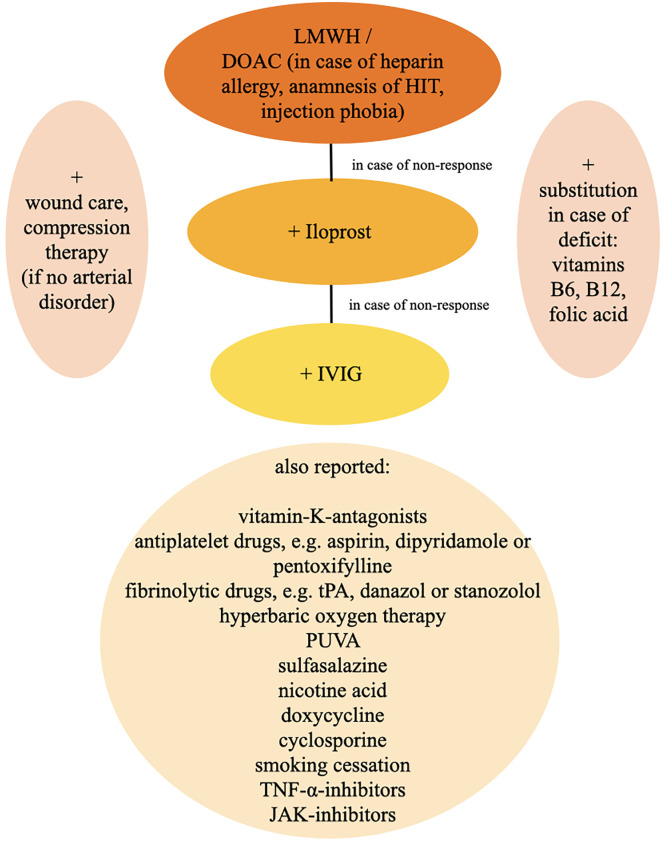
Therapy algorithm. DOAC, direct oral anticoagulants; HIT, heparin-induced thrombocytopenia; IVIG, intravenous immunoglobulins; JAK-inhibitors, januskinase-inhibitors; LMWH, low-molecular-weight heparin; PUVA, psoralen plus UVA; TNF-α-inhibitors, tumor necrosis factor-alpha-inhibitors; tPA, tissue-type plasminogen-activator.

There are many different treatment approaches in LV, however no standardized and evidence-based therapeutic strategies are published. The aim of treatment in LV is an improvement of skin lesions, prevention of relapses and especially a reduction of pain (57). A single therapy approach is not equally effective for all patients, so that several treatment options have to be considered or combined (Figure 10) (21).

Because of the pathophysiological concept of the formation of microthrombi, an anticoagulatory therapy, e.g., with low molecular weight heparins (LMWHs) or direct oral anticoagulants (DOACs) is widely accepted as first line treatment and recommended by the german S1 guidelines (28, 38, 57).

In our clinics we start with a LMWH (i.e., tinzaparin or enoxaparin). LMWHs are most effective, safe and relatively favourable in contrast to other therapy options (13). We recommend a therapeutic dose first (i.e., tinzaparin 175 I.E./kg BW 1x/d, enoxaparin 1 mg/kg BW 2x/d) and a semi-therapeutic dose for maintenance therapy at a stable stage of the disease or after healing of the ulcers. LMWHs are usually well-tolerated and show no drug interaction. Increased risk of bleeding, hematoma, menorrhagia, anemia or local reactions at the injection site can be reported as side effects. Attention should be paid to severe adverse effects like allergic reactions and heparin-induced thrombocytopenia (HIT). In case of impaired renal function dosage must be decreased or treatment is even contraindicated. Regular monitoring under treatment is not necessary. In LV, a successful and prompt response to LMWH was not only reported for adolescents (58), but also for pediatric patients (17, 59).

DOACs are assumed to show a similar effect as LMWHs in the treatment of LV. The so far only registered multi-centre, single-arm, prospective study about rivaroxaban for LV (RILIVA) was recently published (10). 25 patients with LV and a minimum pain score of 40 on the visual analogue scale received oral rivaroxaban for 12 weeks. The initial dosage was 10 mg 2x/d, which was reduced to 10 mg 1x/d, if pain was decreased by 50% on the visual analogue scale. As a backup treatment, subcutaneous enoxaparin 1 mg/kg 1–2x/d could be administered in case of insufficient therapeutical response or exacerbation of pain. During the trial, 5 of 25 patients dropped out of the study. The study showed a significant reduction of median pain. 30% of the patients needed an additional treatment with enoxaparin. 8 treatment-related adverse events were reported in 24% of patients. Weishaupt and coworkers showed with this trial, that rivaroxaban seems to be an effective and safe treatment option for patients with LV.

According to the RILIVA study, we suggest a dosage of 10 mg 2x/d rivaroxaban for initial treatment and a dosage of 10 mg 1x/d in the maintenance phase. Most frequent reported side effects are bleeding, hematoma, menorrhagia, anemia, dizziness, gastrointestinal symptoms, hypotension, skin rash and pruritus. So far, other DOACs such as apixaban were not analyzed in the treatment of LV, but because of equivalent mode of action as direct factor-Xa-inhibitors the effect in treatment of LV is supposed to be similar. We suggest a dosage of 2 × 5 mg daily in the initial therapy with a reduction to 2 × 2, 5 mg daily for maintenance therapy.

The oral instead of subcutaneous application and the absent need of a monitoring of blood values increase the compliance of the patient to DOACs in comparison to LMWHs, although possible side effects, especially increased bleeding tendency, drug interaction, renal and liver impairment have to be elucidated (10, 60). Especially in case of heparin allergy, anamnesis of heparin-induced thrombocytopenia (HIT) or injection phobia DOACs represent an alternative to LMWHs.

**FIGURE 11:**
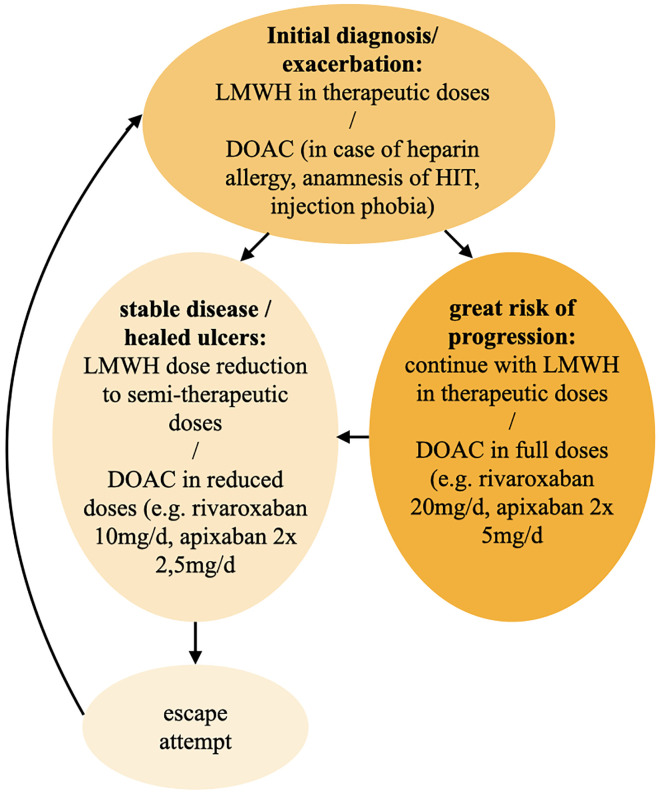
Therapy algorithm for 1st line anticoagulation. DOAC, direct oral anticoagulant; HIT, heparin-induced thrombocytopenia; LMWH, low molecular weight heparin.

Interestingly, therapeutic response to anticoagulation therapy can be accessed rapidly as the patient notices a pain relief within a few days (10, 59). For the initial treatment of LV or in case of exacerbation we recommend a therapeutic dosage of LMWHs or DOACs and continue with this dosage in great risk of progression (Figure 11). After pain relief and healed ulcers and in case of stable disease, a reduction to semi-therapeutic dosages up to an escape attempt is suggested.

In case of an insufficient therapeutical response, we add prostacycline-analoga such as iloprost as an intravenous therapy in a maximum dosage of 20 μg per day over 3–5 days every 4 weeks (61). Dosage is adapted to individual tolerance. Frequently reported side effects under therapy with iloprost contain headache, flush, nausea, emesis and hypotension. Thus, cardiovascular function should be monitored carefully and advanced heart insufficiency must be excluded. In therapy resistant cases we finally add intravenous immunoglobulins (IVIG) in a dosage of 2 g/kg over 2–5 days every 4 weeks. Overall, IVIG is a well-tolerated treatment option in most patients. Possible side effects comprise shivering, headache, fever, dizziness, nausea, vomiting, allergic reactions, arthralgia, hypotension and back pain. In order to detect even very rare adverse effects such as sudden hypotension, anaphylactic shock and transfusion-related acute lung insufficiency (TRALI), infusion rate should be reduced in the initial 30 min of application and can be increased, if well tolerated. Vital signs as well as renal function and blood count must be controlled. IVIG is very effective in the treatment of LV and shows in almost all patients a prompt and sufficient response. Almost all studies report a prompt benefit in >90% of LV patients treated with IVIG (62, 63). However, application should be well considered due to high costs (57, 63–66).

Further therapy options include vitamin-K-antagonists, antiplatelet drugs, e.g., aspirin, dipyridamole or pentoxifylline, fibrinolytic drugs, e.g., tissue-type plasminogen-activator (tPA), danazol or stanozolol (31, 57). Furthermore, there are case reports about a beneficial application of hyperbaric oxygen therapy as well as psoralen plus UVA (PUVA), sulfasalazine, nicotine acid, doxycycline and cyclosporine (31, 57). Moreover, it is presumed, that smoking cessation could have a positive effect for the disease (61). Some authors reported about patients who responded well to therapies with tumor necrosis factor (TNF)-alpha-inhibitors adalimumab and etanercept (67, 68). Recently, positive outcomes upon treatment with januskinase-inhibitors, like Tofacitinib (69) and Baricitinib (70), were reported. Because of often co-existent venous insufficiency a compression therapy for reduction of edema and stimulation of fibrinolysis seems to be effective after exclusion of an arterial disorder (31, 39). An adequate therapy of pain is crucial for impairment of quality of life (9), whereas a pain reduction is often achieved by usage of anticoagulants (71) as well as an appropriate wound care (31). Although an anti-inflammatory therapy with non-steroidal anti-inflammatory drugs (NSAID) is often applied, this therapy failed to show a significant success rate (13).

## Discussion

The present article highlightens the recent findings of current research on LV. The understanding of this still somewhat enigmatic disease has evolved over time and important works have helped to understand, that LV needs to be classified as a thrombotic disease of the cutaneous microcirculation rather than a primary inflammatory vasculitis (1, 3, 15, 17). What remains unexplained up to now is the precise description of the exact molecular mechanism underlying this disease. We have learnt from patient register studies and trials, that pro-coagulatory factors can be identified in about 50% of LV-patients – however, these are heterogenous and thus in the overall description not specific enough (13, 17, 38). What is the specific trigger that tips the physiological balance of continuous fibrin formation and thrombolysis toward thrombotic vessel occlusion? As we observe, that LV is a coagulation disorder strictly limited to the cutaneous microcirculation we need further explanations in how far the cutaneous capillary bed differs from that found in the e.g., renal, hepatic, pulmonary or cerebral microcirculation? What role can be attributed to the altered levels of blood pressure and perfusion velocity that prevail in the lower extremity, with an observed prevelance of LV-ulcerations that are mostly located on the foot and never exceed the knee level (13). This observation clearly points to specific regional conditions leading to reduced blood flow – but what are they exactly?

In terms of differential diagnoses that need to be separated from LV the authors in their treatment centers follow the strategy, that in case of doubt, a hard-facted diagnosis of e.g., antiphospholipid syndrome outweighs the clinical diagnosis of LV rather than diagnosing a LV “secondary” to an antiphospholipid syndrome as other authors suggest (1, 28). Indeed, we observe that there is either a clear diagnosis of a “primary” LV that also responds immediately to treatment or a clinical condition presenting aspects of LV that do not respond adequately to treatment (10) and would thus need additional efforts for finding the right alternative diagnosis. As mentioned above, the fact that LV is strictly limited to the skin helps to separate the impact of *Livedo racemosa* in patients with systemic neurologic disease suffering from Sneddon’s syndrome from those with the same skin lesions affected by LV.

The fact, that there is accumulating evidence and understanding, that LV is a coagulatory disease has significantly influenced the way it is treated over the years. Treatment options that are primarily anti-inflammatory have been used in the past, whereas antithrombotic strategies have evolved over time (58).

Although the exact molecular mechanism that leads to thrombosis is still ignored most authors and guidelines (38) agree, that low-molecular weight heparin is capable of reducing LV-activity (10, 22). Antithrombotic strategies that include fibrinolysis through recombinant t-PA have limitations in their accessibility and might present unwanted side effects hard to manage in an ambulatory setting. With the introduction of novel oral anticoagulants it has been effectively shown in a clinical trial that rivaroxaban is a successful agent in treating LV (10) and was confirmed in subsequent studies (60). The future will show, if the introduction of anticoagulatory drugs might help to further understand LV, if these selectively would not succeed in preventing cutaneous vessel occlusion.

This review is limited by the quality and amount of studies published. There are no randomized, controlled trials on this topic available. Only few prospective studies could be identified. Hence, evidence for diagnosis and treatment of LV is rare. So, even more expert advice is necessary for adequate handling of LV patients.

We conclude that the efforts of all groups in understanding LV have helped to reveal important aspects of the disease, however, we must admit as of now, that there are still huge gaps in the complete understanding of this skin-specific coagulation disorder.

## Data availability statement

The original contributions presented in this study are included in the article/supplementary material, further inquiries can be directed to the corresponding authors.

## Author contributions

All authors wrote the main manuscript text, contributed to the article and approved the submitted version.

## Abbreviations

ADAMTS-13: a disintegrin and metalloproteinase with a thrombospondin type 1 motif member 13
Apo(a): apolipoprotein(a)
C3: complement factor C3
COVID-19: corona virus disease 2019
DOAC: direct oral anticoagulant
HIT: heparin-induced thrombocytopenia
IgA: immunoglobulin A
IgG: immunoglobulin G
IgM: immunoglobulin M
IVIG: intravenous immunoglobulins
LDL: low density lipoprotein
LMWH: low molecular weight heparin
LV: livedoid vasculopathy
MTHFR: methylentetrahydrofolat
NSAID: non-steroidal anti-inflammatory drugs
ox-Lp(a): oxidative form of Lp(a)
PAI-1: plasminogen-activator-inhibitor-1
PAN: Panarteriitis nodosa
PRISMA: Preferred reporting items for systematic reviews and meta-analyses
PUVA: psoralen plus UVA
RILIVA: rivaroxaban for livedoid vasculopathy
SARS-CoV-2: Severe acute respiratory syndrome coronavirus type 2
TF: tissue factor
TFPI: tissue factor pathway inhibitor
tPA: tissue-type plasminogen-activator
TRALI: transfusion-related acute lung insufficiency.
